# Supplementary figures and images for: Meta-analysis of Epstein-Barr virus genomes in Southern Chinese identifies genetic variants and high risk viral lineage associated with nasopharyngeal carcinoma
Source: PLoS Pathog. 2024 May 28;20(5):e1012263. doi: 10.1371/journal.ppat.1012263 (PMC11161099; doi:10.1371/journal.ppat.1012263)

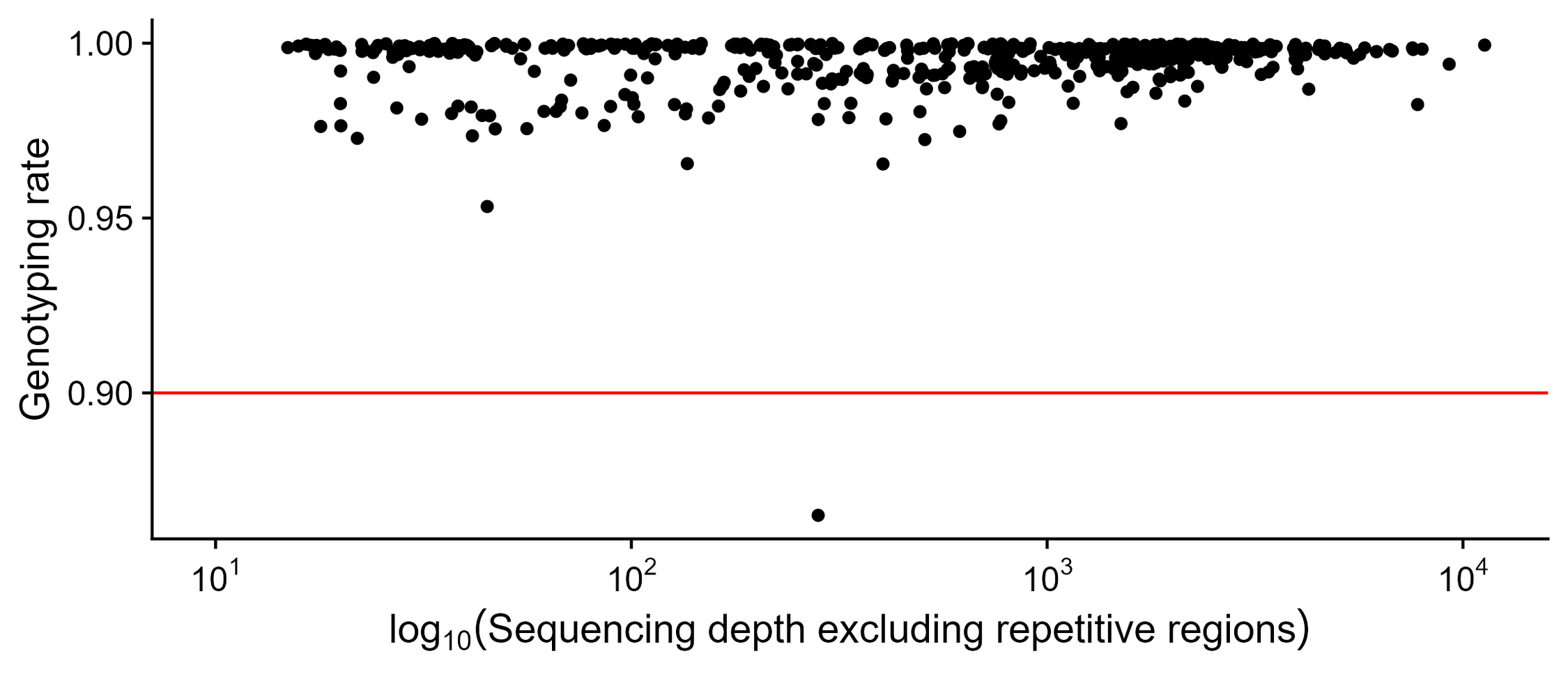

Supplement: S1 Fig — Each dot represents the genotyping rate of a sample. Horizontal red line indicates the genotyping rate threshold at 0.9. (TIF) [file ppat.1012263.s001.tif]

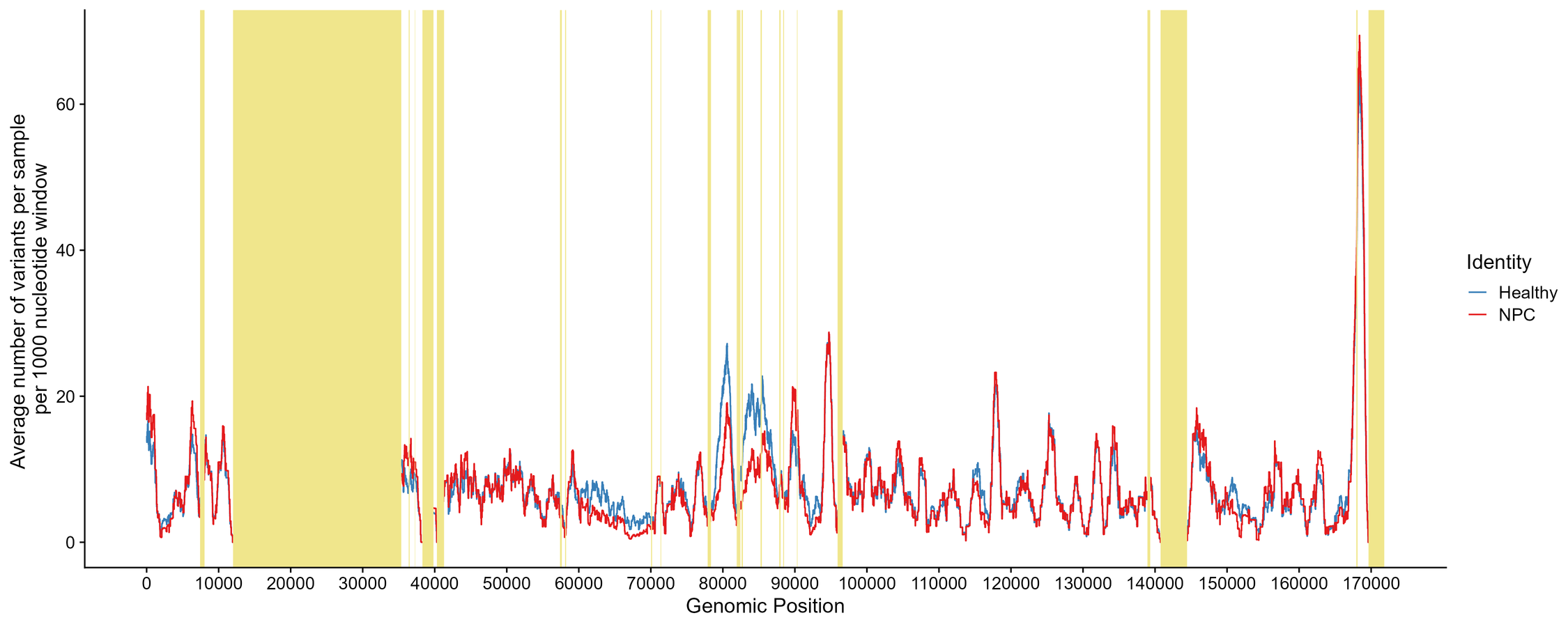

Supplement: S2 Fig — Blue and red lines indicate the variant profiles for healthy individuals and NPC cases respectively. Repetitive regions in the EBV genome are shaded in yellow. (TIF) [file ppat.1012263.s002.tif]

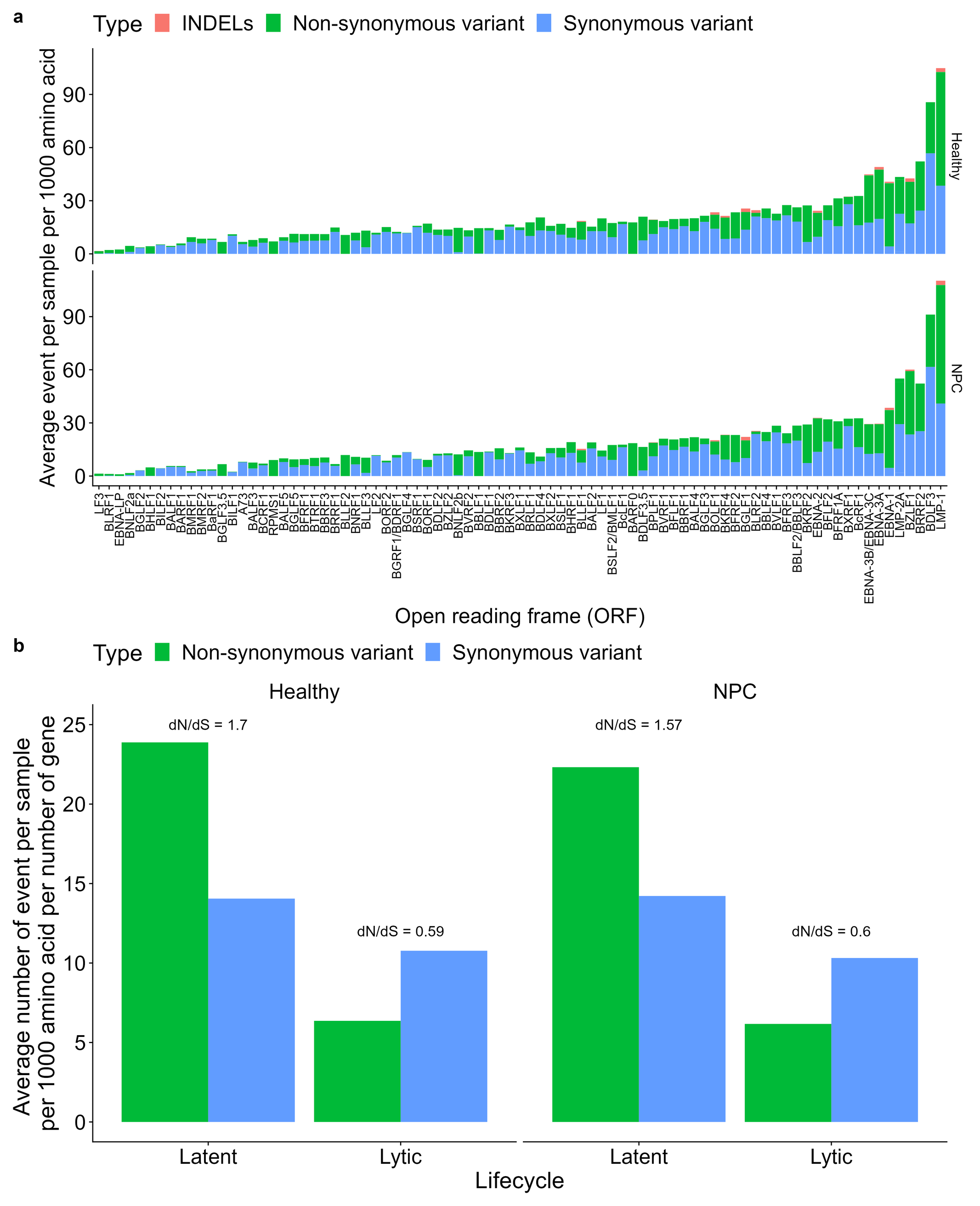

Supplement: S3 Fig — (a) The number of synonymous and non-synonymous variants in each open reading frame (ORF) is arranged by the total sum of variants in the ORF. The numbers are normalized by the number of individuals in each cohort and by the gene length per 1000 amino acids. (b) The number of synonymous and non-synonymous variants in latent and lytic genes. The numbers are further normalized by the total number of genes classified as latent/lytic. dN/dS, ratio of non-synonymous variant to synonymous variant. (TIF) [file ppat.1012263.s003.tif]

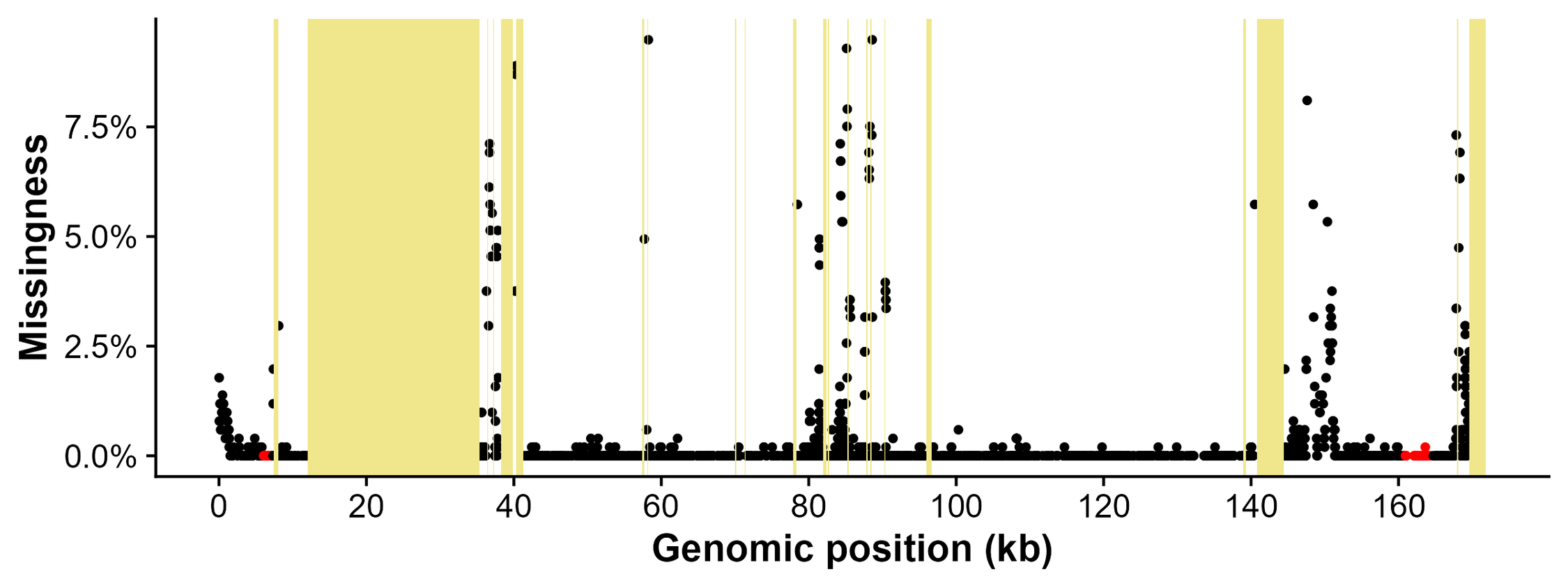

Supplement: S4 Fig — Each dot represents the percentage missingness of a variant. Repetitive regions in the EBV genome are shaded in yellow. Variants spanning the upstream of EBER1 (immediately downstream of BNRF1) to the downstream of EBER2 (immediately upstream of OriP), and BALF2 are colored in red. (TIF) [file ppat.1012263.s004.tif]

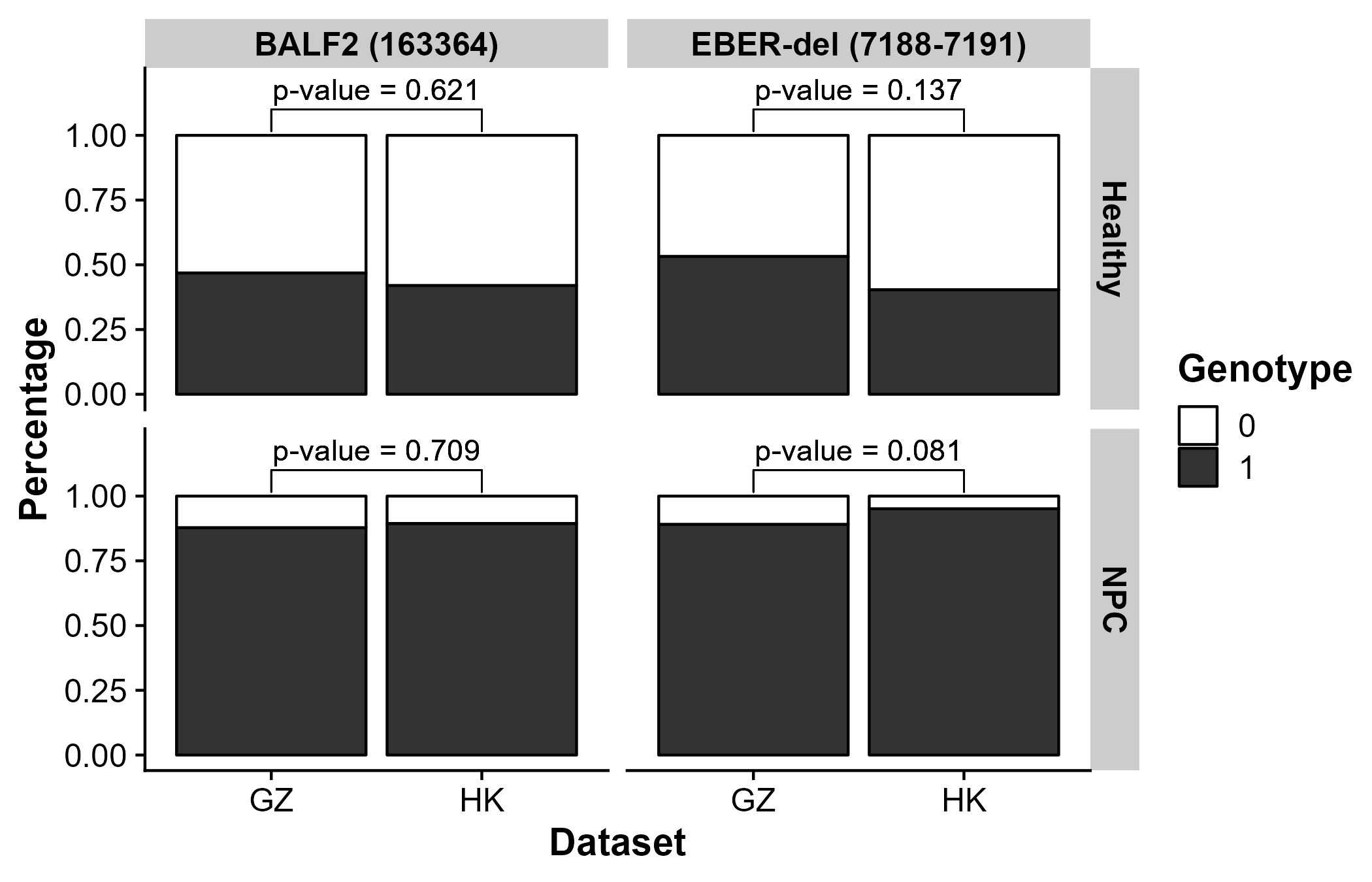

Supplement: S5 Fig — The two columns indicate the distribution of genotypes in the BALF2 risk loci and EBER-del respectively. The two rows indicate the distribution of genotypes of healthy controls and NPC cases respectively. P-values from the Fisher’s exact test of allelic counts are shown on top of each bar. (TIF) [file ppat.1012263.s005.tif]

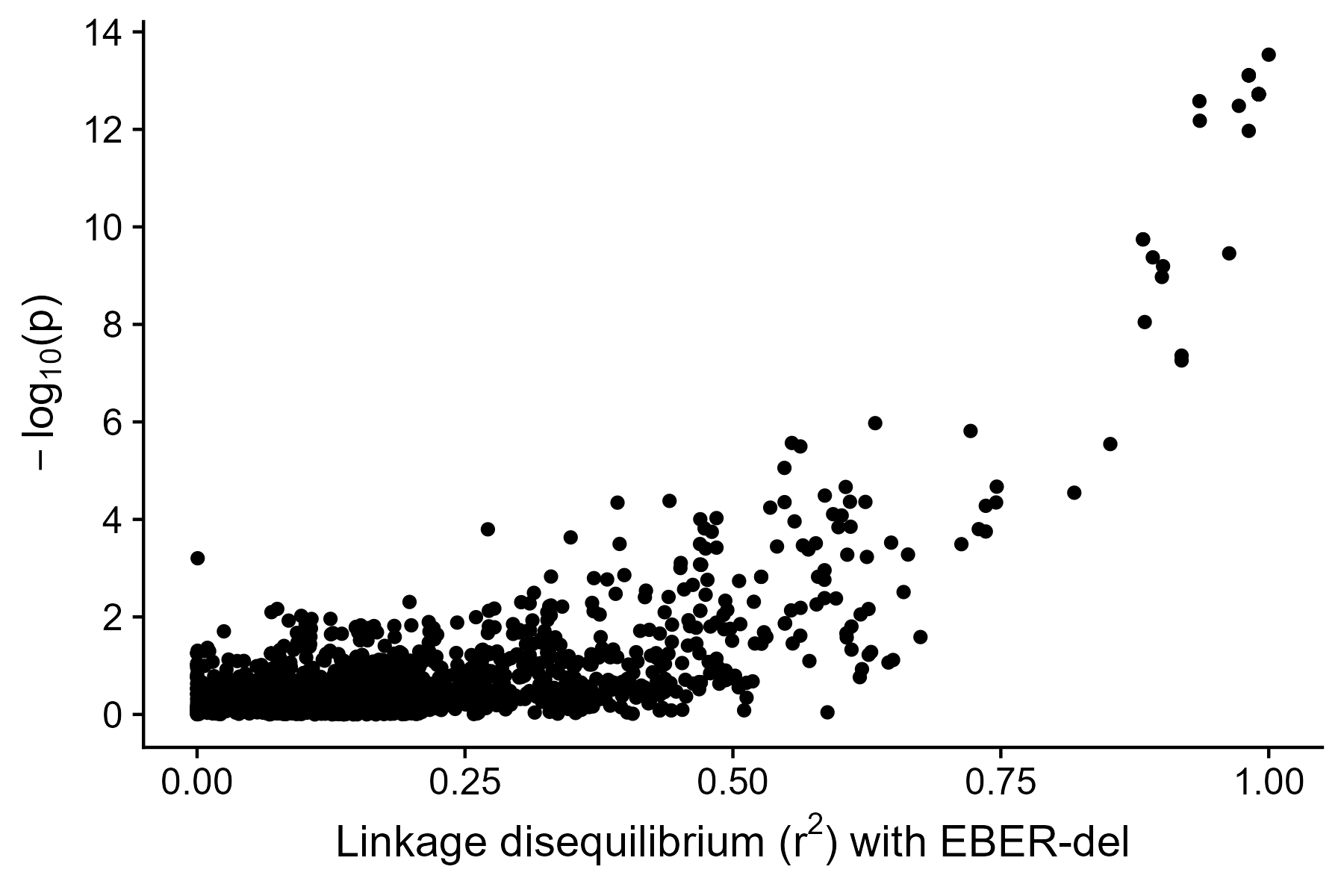

Supplement: S6 Fig — Each dot represents a variant in the meta-analysis of GWAS. (TIF) [file ppat.1012263.s006.tif]

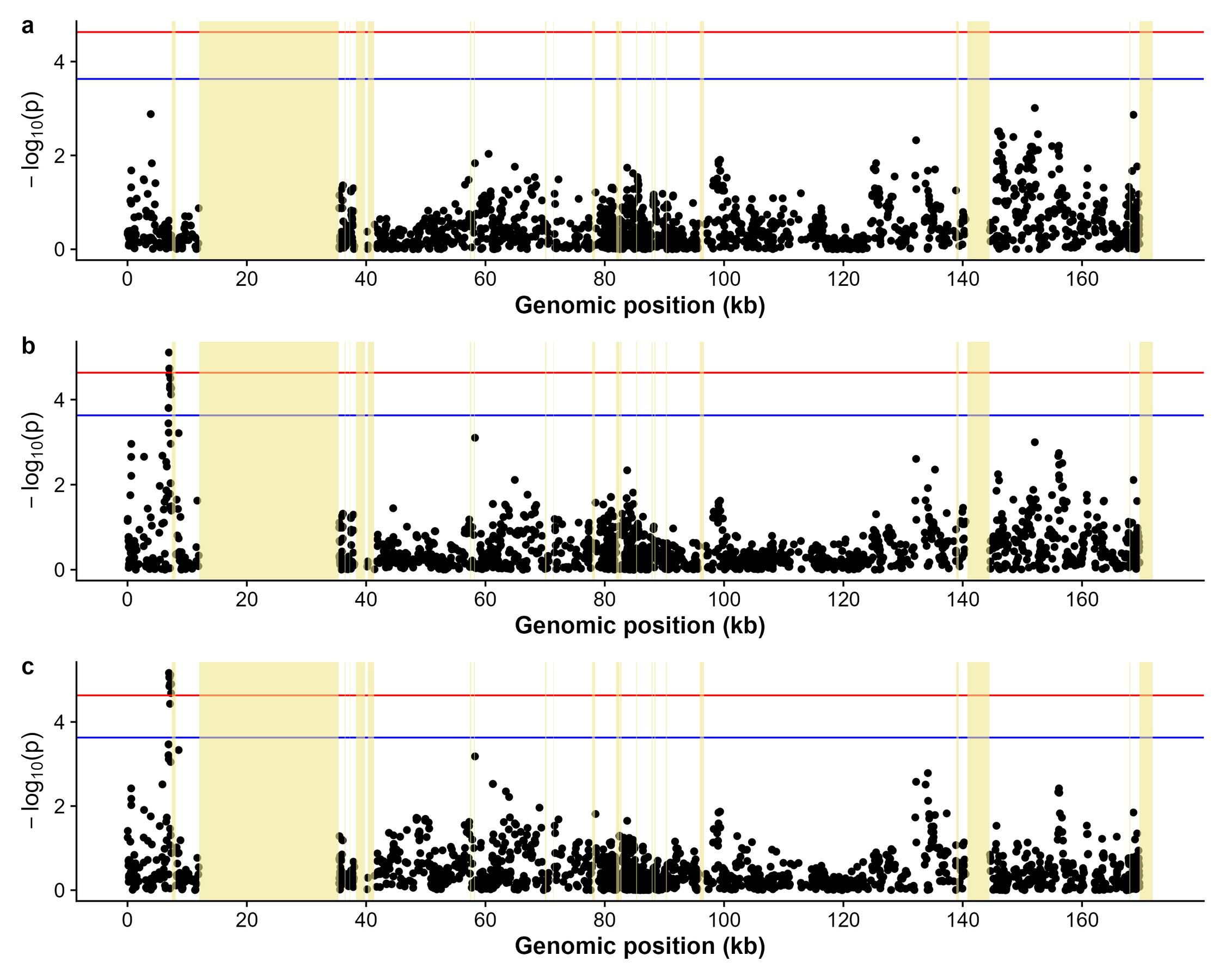

Supplement: S7 Fig — (a) Conditional on EBER-del. (b) Conditional on variant 163364. (c) Conditional on variants 148489, 150028, 151822 and 152087. Repetitive regions in the EBV genome are shaded in yellow. Red line: Bonferroni-corrected genome-wide significance at 2.33×10^−05^; blue line: permuted genome-wide significance at 2.35×10^−04^. (TIF) [file ppat.1012263.s007.tif]

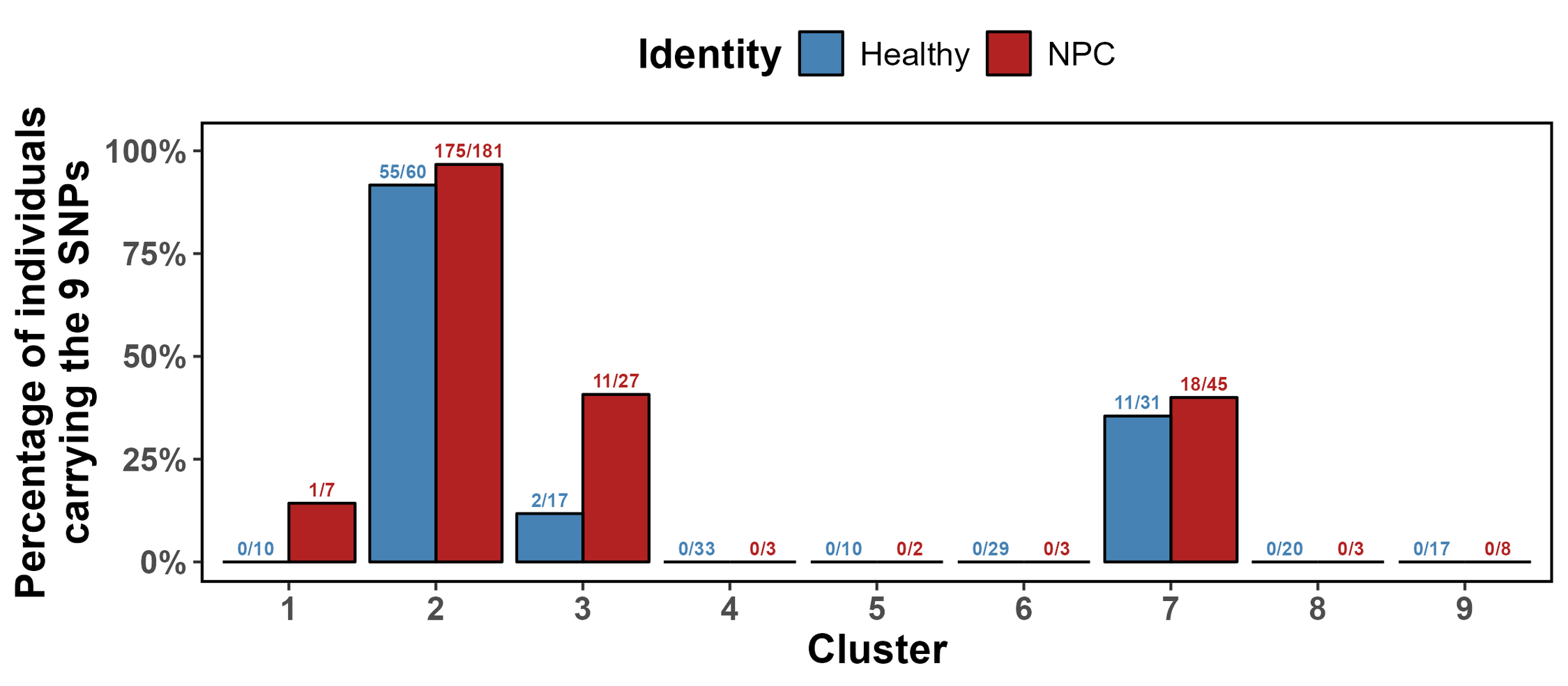

Supplement: S8 Fig — The number of cases/controls are labelled on each bar chart, colored according to their identity. (TIF) [file ppat.1012263.s008.tif]

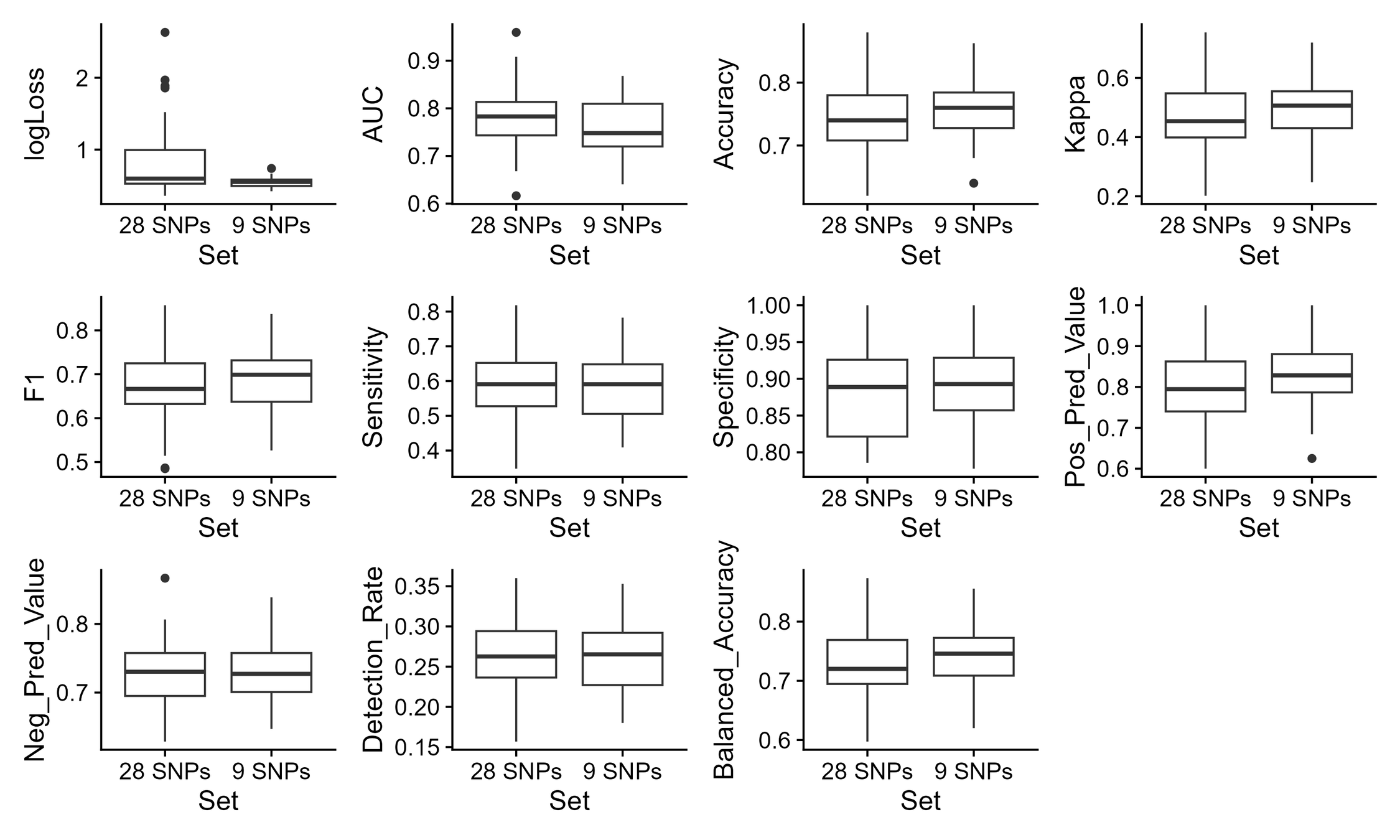

Supplement: S9 Fig — Each boxplot represents the distribution of one performance metric, generated by a ten-fold cross-validation with five-repeats. (TIF) [file ppat.1012263.s009.tif]
