# Supplementary material for: Meta-analysis of Epstein-Barr virus genomes in Southern Chinese identifies genetic variants and high risk viral lineage associated with nasopharyngeal carcinoma
Source: PLoS Pathog. 2024 May 28;20(5):e1012263. doi: 10.1371/journal.ppat.1012263 (PMC11161099; doi:10.1371/journal.ppat.1012263)
Supplement: Attachment — Submitted filename: Point_to_point_response_PLOS_Pathog_202404253.docx [file ppat.1012263.s017.docx]

# Point-to-point response for “Meta-analysis of Epstein-Barr virus genomes in Southern Chinese identifies genetic variants and high risk viral lineage associated with nasopharyngeal carcinoma” by Ka Wo Wong et. al.

**Part I - Summary**

Please use this section to discuss strengths/weaknesses of study, novelty/significance, general execution and scholarship.

***Reviewer #1****: Wong et al add a new set of case-control samples to two existing EBV genome datasets (one from their lab), and then extend existing SNP mapping to identify other risk variants. Importantly, this study is (to my knowledge) the first to extend these analyses to haplotype mapping, which is essential for moving towards the use of viral genetics to facilitate targeted screening. Doing so, they identify what appears to be a high risk haplotype.*

*While the goal and approach is generally right, I have two substantial criticisms that should be addressed before these results can be considered robust and informative. This essentially revolves around the way the analyses have been executed (or in some cases perhaps explained), in particular the lack of robust approaches to control for the unequal sizes of control and case groups from the two geographical areas being studied. Since I am not well versed in genetic methods, I cannot tell how difficult these would be to address.*

**Author’s response:** Major issues are addressed in subsequent sections of the point to point response.

***Reviewer #2****: Nasopharyngeal carcinoma (NPC) is prevalent in Southeast Asia. It is thought that environmental factors, human genetic variation, and genetic diversities of EBV contribute to EBV-associated NPC. Wong et al identified 38 single nucleotide polymorphisms (SNPs) in 61 NPC patients carrying high-risk EBV lineage which encodes a G6944A variant at the upstream of EBER2. However, if these SNPs indeed correlate with NPC incidences, they should be aligned to the sequence of M81 strain which was derived from an NPC patient rather than the B95.8 strain (Figure 5). All SNPs in the cluster 2, 3 and 7 should be listed, and create a Venn diagram of intersections for all SNPs. To conclude these G6944A and 38 SNPs can be implicated in the development of NPC, EBV strains from areas other than Southeast Asia should also be analyzed to ensure the specificity of these variations and SNPs.*

**Author’s response***:* Thank you very much for your comment. In our study, we are using the method of “reference mapping” to analyse the short-read sequencing data from NGS. Under this context, a reference genome serves as a guide on how to piece the short reads together. The reference genome will have no role in the actual genotypes being called. B95.8 is a commonly used reference genome in the field where sequence annotations are mostly based on this reference. Using B95.8 as the reference genome is the standard way of performing analyses. Secondly, a Venn diagram showing the numbers of SNPs in Clusters 2, 3 and 7 is shown here. The Venn diagram showed that Cluster 2 and Cluster 3 are more similar with each other than Cluster 7 (see Venn diagram attached in this response letter). Finally, EBV genomes derived from NPC tumors outside of South China are currently lacking. Hence, we would not be able to perform sequence analysis from other areas to confirm the role of the variants.

**Part II – Major Issues: Key Experiments Required for Acceptance**

Please use this section to detail the key new experiments or modifications of existing experiments that should be absolutely required to validate study conclusions.

Generally, there should be no more than 3 such required experiments or major modifications for a "Major Revision" recommendation. If more than 3 experiments are necessary to validate the study conclusions, then you are encouraged to recommend "Reject".

***Reviewer #1****: My major issue is the way the Hong-Kong and Guangzhou datasets were combined. The HK data set had a case: control ratio of 2:1 and the GZ data was a ratio of 1:2.5. Therefore any statistical analysis of SNP enrichment will also identify SNPs that statistically differ between HK and GZ. The authors present PCA that they claim shows the two populations are equivalent, but this is both not a suitable test for this (PCA includes only a subset of variation) and unconvincing (HK is significantly enriched in the bottom left quadrant of the PCA fig 2b, even if this simply reflects the more ethnically diverse history of HK). Therefore, the data would be much more robust if the authors used a two factor statistical test (maybe ANOVA) combining sampling site with case-control. It would also be helpful to present sample site-split case control data sets (and present proportions rather than case numbers) as supplementary to help validate data such as in figures 4b and 5b.*

**Author’s response:** Thank you for the detailed comment. We acknowledge the limitation of PCA in assessing the similarity between populations. We employ PCA to visualize the structure of EBV genetic data. To supplement the PCA, we also conducted a nucleotide diversity analysis using the whole EBV genome sequences of each sample of HK and GZ cohorts (Fig 2c). This demonstrated that EBV genomes between HK and GZ samples (both NPC cases and healthy controls) are highly similar (lower the nucleotide diversity, higher the similarity). We also demonstrated that the pairwise nucleotide diversity among GZ and HK cases and controls are highly similar. We also included EBV genomes from distant location (Kenya) to show the nucleotide diversity patterns of dissimilar genomes. All the above data are shown in Fig 2c. Secondly, regarding your comment on the enrichment of HK samples in the bottom left quadrant of the PCA plot (Fig 2b), we would like to clarify that only a small fraction of the HK samples (6 out of 272; 2.2% of all EBV Type I HK samples) are located in this quadrant (defined as PC3 < - 0.15). And one sample from GZ (1 out of 187; 0.53% of all EBV Type I GZ samples) is located in this quadrant. The small percentages would not represent significant difference between HK and GZ cases. Thirdly, to better address the problem of imbalance of case-control ratio in the dataset, we have now performed **meta-analysis** of two independent GWAS (the first GWAS includes HK samples only and the second GWAS includes GZ samples only) controlling for population structure in the logistic regression model (now shown in Fig 3). The most significant region associated with NPC is still around EBER with secondary signals locating at the 3’ end of the linear EBV genome. The most significant variant is the 4-bp deletion downstream of EBER2 instead of G6944A as stated in the first draft. One of the explanations for this is the strong linkage disequilibrium within the variants of EBERs making it very difficult to pinpoint the exact causal variant in GWAS. This is also the reason we are proposing the high-risk lineage that is associated with NPC instead of a single variant. We also looked at the allele frequency of the top GWAS signals reported previously (EBER-del and BALF2 163364) and found that the frequency of the risk allele in HK/GZ case and controls are similar (Fisher’s exact p-values > 0.05, S5 Fig). Similar analysis was also performed for the downstream haplotype analysis where the percentage of case/controls carrying the risk haplotype is similar in HK/GZ samples (Fig 4c). In addition, the original bar charts of Figs 4b and 5b were revised to show percentage on the y-axis instead of count number (now Fig 5b and S9 Fig).

***Reviewer #1****: My second issue is around how the haplotype analysis was conducted (or explained). The study picked - apparently arbitrarily - 8 SNPs for their initial analysis. If this was all the mis-sense SNPs, then this makes the assumptions that any important SNPs will be coding (despite the fact that the top hit - EBER2 promoter) is clearly non-coding. The paper then produces a maximum risk haplotype of 38 SNPs using an agnostic method. In my view this has been done the wrong way around, and the agnostic method has not been leveraged sufficiently. The haplotype selection process is a bit opaque, and there is a lack of clarity of what percentage of cases have vs do not have this haplotype, and how to leverage the haplotype (and more importantly deviations from the haplotype) to inform predictive diagnosis. If this could be incorporated, it would maximise the value of the paper.
What would be really valuable to the field would be to know which are the biggest contributors to NPC within this high risk haplotype. I have seen cumulative contribution analyses when attempting to reduce gene signatures associated with a disease to the minimum size for maximum predictive value. My colleague tells me this is a machine learning strategy called “feature selection” (specifically forward selection - adding them one by one and checking the performance metrics [significance or odds ratio] each time), and/or using logistic regression or decision trees. I think this approach to selecting the most predictive SNPs would be an agnostic approach to both identifying the most predictive SNPs, and potentially identifying SNPs that would be most important for future biomarker studies. Such a refined set of SNPs could then be more rationally used in the analyses described in figure 4/tables 1&2. This kind of insight would make the paper both more robust (in terms of avoiding investigator assumptions) and more impactful in focusing future research.*

**Author’s response:** Thank you for the detailed comments. We acknowledged the problems in the original analyses pointed out by the reviewer. We now performed two independent analyses that involve multiple variants. (1) The first analysis is driven by the results of the meta-analysis of GWAS where ALL variants (SNPs and INDELs; irrespective of coding or non-coding) that passed the permuted p-value cut-off (total 56 variants) were included in the construction of the haplotypes. The haplotype analysis likely gives a list of variants that are potentially causal to NPC as they are driven by the p-values of the meta-analysis. To better visualize the data, we included a new Fig 4 to describe the results of the haplotype analysis, and the original Tables 1 and 2 were replaced by S3 and S4 Tables. (2) The second analysis is driven by the clustering analysis of phylogenetic tree and population structure where the most common SNPs in each population cluster are curated and only the SNPs unique to the high-risk clusters (clusters 2, 3 and 7) are extracted, yielding detailed characterization of the genetic structure of the high-risk EBV lineage. As suggested by the reviewer, we further incorporated two methods, LD clumping and a feature selection algorithm (XGBoost), to identify the SNPs that have the best predictive power for NPC. In this way, we successfully reduced 38 SNPs to 9 SNPs that have comparable performance in predicting NPC (shown in Fig 6) which are validated by a 10-fold cross-validation with five-repeats (the distribution of performance metrics across the iterations is shown in S9 Fig).

***Reviewer #1****: Third, this sort of study is highly dependent on the multiple sequence alignment (MSA) used by the investigators. This MSA should be directly available, either from a data repository or as supplementary data file, to help others to validate the study.*

**Author’s response:** We apologize for any confusion in the first draft. The method for identifying the (original) 38 SNPs is not by MSA but by a custom script (originally described under Method section “Phylogenetic analysis and clustering” of the first draft). We have now written a new Method section “Identification of single nucleotide profile that characterised the high-risk EBV lineage (lines 438-445) to describe the process in detail and the script and raw data necessary to reproduce the 38 SNPs are uploaded on GitHub (<https://github.com/AlanC-lab/Integrated_EBV_genomes_NPC_South_China/>) as the data repository.

**Part III – Minor Issues: Editorial and Data Presentation Modifications**

Please use this section for editorial suggestions as well as relatively minor modifications of existing data that would enhance clarity.

***Reviewer #1****: I also have a number of minor comments below that I compiled as read the paper, that should be considered or addressed, for clarity, accuracy, precision, or methodological robustness. This includes some references to the major issues mentioned above.*

*Abstract:*

*22 - clarify multiple SNPs (why variants?) forming a high risk haplotype? Or independent linkages?*

**Author’s response:** Non-synonymous variant is a common term to describe mutations that lead to a change in coding amino acid. Apart from SNPs, INDELs also have strong LD with the top GWAS signal. Therefore we believe the term “variant” might be better than “SNPs”.

*26 Panel of SNPs: say something about interdependency/recombination/haplotype. Multiple mentions of panel (summary) seems redundant?*

**Author’s response:** By the method of LD clumping, we believe the concern on interdependency is resolved. Also, we have rewritten this part to avoid redundancy (lines 25-26).

*61 Line mentions preponderance for Type I in NPC without context of background type1:type2 prevalence in the high risk population. Comment on relative frequencies in South China.*

**Author’s response:** Included references stating Type I EBV is prevalent worldwide including South China (lines 60-61; ref 18-23). Based on our dataset, 195/227 (86%) healthy controls carry the Type I EBV genomes while 267/279 (96%) of NPC cases carry the Type I EBV genome.

*64 - endemic regions: terminology confusing in the context of endemic BL. Regions of high NPC prevalence?*

**Author’s response:** Revised to “endemic regions of NPC” (lines 64-65).

*66. Ref 22 is much more complicated than the interpretation here: the paper made a chimeric M81/B95-8 EBV that likely resulted in mismatches between polymorphisms in N- and C-terminus, it may be these mismatches that reduced EBNA-1 function, rather than this polymorphism alone. It therefore may not reflect the real biology in circulating EBV strains.*

**Author’s response:** The text is re-written to avoid any mis-interpretation as suggested by the reviewer (lines 66-68; ref 31).

*70/71 - ref 28 shows SNPs common to China/indonesian EBVs (NPC-high regions) having altered miRNA expression profiles: it does not test NPC-association within those regions, so the claim should be toned down as a geographical link, rather than a disease link.*

**Author’s response:** The original sentence commenting on disease link is now deleted.

*78-80 - when describing the EBER2 polymorphisms, it may be useful to the reader to include position relative to EBER1 (one of the two polymorphisms will be between EBER2 and EBER1) - eg “less than 20 nt upstream of EBER2”. Also, T6999G (fromhttps://doi.org/10.1099/jgv.0.001728) position is within EBER2 on my NC7605 map. Please clarify (and clarify whether this is the functional EBER2 SNP characterised by the Delecluse (Li et al 2019) and Tibbetts (Wang et al 2022) labs).*

**Author’s response:** We apologize for the typo. T6999G is indeed within EBER2 (line 79), and it is the SNP characterised by the mentioned papers. Cited the two papers (Li et al 2019 and Wang et al 2022) when discussing the function of EBER2 in DISCUSSION (lines 285-287, ref 42, 43).

*94 - explain what “279 and 227” this was confusing: suggest attach the numbers to the descriptors. case vs control? [controls are assymmetrically matched?]*

**Author’s response:** Now stated 279 cases and 227 controls.

*Statistics: How does the study mathematically handle the unequal sizes of cases and controls from each study? HK1 is 142+61/62+31; GZ is 61/156. How do you know your study is not showing differences between HK and GZ, because of this skew?*

**Author’s response:** This point is addressed in Author’s response in Part II reviewer #1. In brief, we performed meta-analysis of GWAS, reported the risk allele frequencies of the two regions and reported haplotype frequency of the two regions. All the results show no signs of skewing based on the regions.

*208 - Statistics conducted in R is insufficient information (unless the individual statistical tests are defined throughout the paper).*

**Author’s response:** The statistical tests are now described under each sub-section of Method. For example, the logistic regression for GWAS and haplotype analysis is described in “Genome-wide association tests (GWAS), meta-analysis of GWAS and haplotype analysis” (paragraph from line 396). The same applies to PCA (paragraph from line 390), phylogenetic tree (paragraph from line 424), population structure analysis (lines 432-434) and feature selection algorithms (paragraph from line 447). In addition, we have added the description of using the Fisher’s exact test for comparing the genotypes across dataset under “Statistical analysis” (paragraph from line 461).

*233-239 - Principal components are generated de novo for every data set, so the implication that geography falls in PC2 or 3 (line 235-6) is not valid. Indeed, just looking at the PCA, you might (by the same logic) conclude that the NPC and non-NPC samples also are not different from each other. And looking at the data, there are no GZ samples (except one NPC) in the bottom part of the PCA. So I feel that the geography in the samples is not fully resolved. A more robust statistical test is required.*

**Author’s response:** We agree that PCA are generated de novo for every dataset. This is more a quality control step to ensure at least the PCA cannot detect any clustering based on HK/GZ. Removed the sentence in lines 235-236 of first draft. To resolve the geographical problem, we performed a pairwise nucleotide diversity analysis to show that samples from South China are highly similar to each other. And we also investigated genotype frequencies split by HK/GZ. This point is addressed in detail in Author’s response in Part II reviewer #1.

*244 - ‘two cohorts’ - is this NPC/ctrl or HK/GZ?*

**Author’s response:** Revised to “between NPC cases and controls” (line 152).

*250 - top association was replicated - was this replication strengthened, weakened or similar, given the additional samples (we would expect p to become even smaller with larger n).*

**Author’s response:** The replication was strengthened. The original report of EBER-del in the IJC paper has a p-value of 1.91×10^−07^, we now have a p-value of 2.94×10^−14^ (S3 Table and Fig 3).

*254 - the non-replication of 163364 warrants additional scrutiny to explain the difference: is your re-calling the sequences changing the frequency of this SNP? Is the prevalence of this SNP different between HK and GZ samples? Or was this just skewed by the low control number in GZ data set the first time around?*

**Author’s response:** The prevalence of 163364 between HK and GZ samples is similar. Strong LD of 163364 and EBER region as indicated in the paper. We have included S5 Fig to confirm the allele frequency of the 163364 and EBER-del in different datasets. Fisher’s exact test was performed on the allelic count of HK and GZ per case/control and no statistically significant result was found.

*267 - variants within R2 0.6-0.8 - this seems to be a different analysis from that in figure 3: Narratively the analysis that gives the degree of association should like to the p value measure in figure 3, or the figure indicating R2 values should be more prominent.*

**Author’s response:** Deleted the original section in the RESULTS. New analyses are performed. Included conditional analysis on EBER-del and 163364 to address the issue of high r2 with top GWAS signal (S6 and S7 Figs). Added a new paragraph describing these new results (lines 181 – 186).

*Overall - not sure I follow the logic of the narrative - surely if you are looking for driver risk alleles other than EBER, you want to investigate SNPs with high P value but low linkage, to maybe identify alternate haplotypes?*

**Author’s response:** Based on the current meta-analysis of GWAS, we see that multiple variants have moderate/strong linkage with the top GWAS signal in the EBER region that at the same time have low p-values, indicating that EBER region will not be the sole player to contribute to NPC. We looked at the haplotypes constructed by ALL variants that passed the current p-value cut-off in order to identify a list of candidates that are potentially causal to NPC.

*277-295 - a haplotype-based analysis is absolutely the right think to do, but the selection of the haplotype seems entirely subjective, and the basis for choosing these SNPs is unclear. Is it reasonable to select only coding mutations, as other (regulatory) changes could be more important but much harder to identify.*

**Author’s response:** Please refer to detailed comment in Author’s response in Part II reviewer #1. In brief, we included all variants that passed our p-value cut-off for the construction of haplotypes in the new analyses to minimise the subjectivity.

*Table 1: REF and ALT may not be the best annotation - REF and NPC or REF/CHN (for China) may be better, since you are defining NPC-associated variants. In a table of all variants, then the “ALT” makes a bit more sense, although it is possible that a REF allele has higher association with NPC than ALT (where the OR is <1, presumably).*

**Author’s response:** Revised to “Risk” allele as in S3 Table.

*Table 2 is the crucial data set that shows a really strong enrichment - again I would prefer a supplemental version looking at the haplotype frequencies in the geographically separated data sets (GZ vs HK). However it is very hard to ‘read’ the haplotypes: could you have all the haplotypes labelled as bold (or coloured) for risk-allele and non-bold as no-risk allele. Then it is much easier to see both the number and structure of risk alleles.*

**Author’s response:** The haplotype is now presented as a heatmap with red showing the presence of the NPC risk allele and grey showing the non-risk allele per position (shown in Fig 4a). The haplotype frequencies in the geographically separated data sets are shown in Fig 4c. The exact genotypes of the haplotypes are listed in S4 Table.

*I think it might also be useful to have a separate analysis (including rare alleles) that shows OR and/or p-value for different numbers of risk alleles from your list. Or if the haplotypes are more structured (showing recombination between two high and low risk haplotypes) then the total length of the risk haplotype.*

**Author’s response:** Since most of the NPC cases carry the risk haplotype, we do not think that expanding the analysis to include rare haplotypes will be helpful. A total of 145 haplotypes are derived from 56 variants and 140 of them have less than seven individuals per haplotype. In this revised haplotype analysis, we already included all “risk” alleles (all of them have p-value smaller than our permuted threshold) identified by the meta-analysis of GWAS.

*In the 16 non-risk haplotype NPC cases, are there any risk-linked SNPs in common? Could these be assessed for being part of the haplotype, or an independent risk locus?*

**Author’s response:** In the revised haplotype analysis, we no longer identified a distinct “non-risk” haplotype. We believe all haplotypes other than the “risk haplotype” are non-risk. We have performed conditional GWAS on all the four sites of haplotype III that are distinct from those of the risk haplotype, the result did not demonstrate these sites to confer independent effect on NPC (S7c Fig, lines 197-199).

*Thought: are there any CTL epitopes (esp in Chinese HLA) linked to the SNPs? Or even DRiPs associated with silent polymorphisms? [may not be possible to answer this]*

**Author’s response:** We do not have these data.

*Figure 4/317-324 - I am not clear how the clusters have been defined: They do not seem to make sense in the context of the phylogenetic tree (with cluster 3 being scattered among several parts of the tree) and the NPC risk-alleles. Perhaps it would have been more useful to cluster only using SNP positions that were defined as associated with NPC at some threshold, and place that in the wider phylogeny of the virus? Ether way, the authors need to explain the rationale behind their particular choice of clustering algorithm, as the results are hard to understand.*

**Author’s response:** We use rhierBAPS for population clustering which can generate discrete population cluster (cluster 1 through 9) based on genetic similarity of the EBV genomes. Minor differences between the phylogenetic tree and the population structure clustering analysis is still present. In the phylogenetic tree, we are using the whole EBV genomic sequences with consideration of the nucleotide substitution rate. But the population structure clustering algorithm by rhierBAPS, uses SNPs as the only input. Phylogenetic tree and population structure clustering analysis yield data that largely agree with each other supporting accurate clustering of 506 samples of this study. As suggested, we tried clustering using a subset of SNPs only but the resolution is not as good as using the whole EBV genome.

*Figure 4b is not a great visualisation of the data - surely much better to use paired bars, with each bar indicating the % of cases or controls. This would offer a much easier to interpret visual representation of cluster enrichment in one or the other group.*

**Author’s response:** The original Fig 4b is now revised and the data are now shown in Fig 5b.

*Line 337 - the key observation (AUC value for ROC) is not easy for the lay reader to understand - can you explain what this means? Is there a positive predictive value or negative predictive value you can describe? How effectively would screening for these SNPs be able to assess NPC risk? Perhaps offer some calculations that indivcate the utility of these SNPs, and would also be useful to graph them by increasing value, to identify minimum SNP usage to maximise predictive value.*

**Author’s response:** The AUC of the ROC represents the ability of the model to correctly classify positive and negative observations with an AUC of 1 indicating perfect classification and an AUC of 0.5 suggesting no better accuracy than random chance. We showed that the performance (including PPV and NPV) in predicting NPC using 28 SNPs and 9 SNPs are comparable (slightly better using 9 SNPs) in Supplementary Figure S9. We add Figures 6a and 6b which illustrates genotype of 9 SNPs and ROC with stated AUC, respectively.

*Line 347 - 6999 within EBER2?*

**Author’s response:** Yes, corrected. Sorry for the typo (line 285).

*Line 356 - may be useful to discuss weighting of the different SNPs within EBER2 as these might affect functions of the molecule (cf papers from Delecluse (ref 57) and Tibbetts (Wang 2022) labs).*

**Author’s response:** Due to high LD within the EBER2 variants, will be difficult to assign weighting of the SNPs.

*Line 395 - if you wish to challenge the findings of another paper, then you should show the analysis. Additionally, any screening approach should use a set of minimally linked genes tfor their screening strategy (while defining which other SNPs the one in the screen is also a proxy for by linkage). So the fact that the previous set is not linked is not inherently problematic (though a lack of weighting for association with NPC probably is a problem!).*

**Author’s response:** We now delete the sentence (line 394-395 of first draft).

*395-7 I suggest potential heterogeneity of EBV (multiple strains in a person) also makes this approach problematic as you cannot be sure that the virus in the NPC is the same as the predominant strain in blood.*

**Author’s response**: Agree with the reviewer that the methodology and application of EBV data in NPC screening protocol needs to be tested in future studies
