# Supplementary material for: Meta-analysis of Epstein-Barr virus genomes in Southern Chinese identifies genetic variants and high risk viral lineage associated with nasopharyngeal carcinoma
Source: PLoS Pathog. 2024 May 28;20(5):e1012263. doi: 10.1371/journal.ppat.1012263 (PMC11161099; doi:10.1371/journal.ppat.1012263)
Supplement: Attachment — Submitted filename: Point_to_point_response_PLOS_Pathog_20240507.docx [file ppat.1012263.s018.docx]

# Point-to-point response for “Meta-analysis of Epstein-Barr virus genomes in Southern Chinese identifies genetic variants and high risk viral lineage associated with nasopharyngeal carcinoma” by Ka Wo Wong et. al.

***Reviewer****: The authors seem to recognize that their analysis requires them first to identify which strain of EBV they consider (type I or type II) and then identify sequence variants in their isolates from each strain that differ form their type I or type II reference genome. However, their description of their analysis is unclear. For example, they write: " To determine the EBV type of our samples, an EBV Type II BAM file was generated by an alignment of the Type II EBV reference genome AG876 (GenBank accession number: NC_009334.1) to the Type I EBV reference genome. The BAM file for each sample was piled up together with the Type II EBV reference BAM file, and variants were called by BCFtools (version 1.13). Nucleotides that were identical in both Type I and Type II EBV genomes were ignored." This description does not make sense and needs to be corrected so that a reader can understand the steps in the authors' analysis to ensure that it does make sense.*

**Author’s response**: The corresponding method section in lines 380 to 385 of the revised manuscript was re-written to clarify the steps in classifying Type I or II EBV genome. The sentence “Nucleotides that were identical in both Type I and Type II EBV genomes were ignored” is deleted and replaced by “Variants were subsequently called by BCFtools including only multi-allelic SNPs.” The above should address the concern raised by the reviewer and clarify the steps performed to ascertain the EBV type.
